# Lipid profile changings after switching from rilpivirine/tenofovir disoproxil fumarate/emtricitabine to rilpivirine/tenofovir alafenamide/emtricitabine: Different effects in patients with or without baseline hypercholesterolemia

**DOI:** 10.1371/journal.pone.0223181

**Published:** 2019-10-11

**Authors:** Lucia Taramasso, Antonio Di Biagio, Niccolò Riccardi, Federica Briano, Elisa Di Filippo, Laura Comi, Sara Mora, Mauro Giacomini, Andrea Gori, Franco Maggiolo

**Affiliations:** 1 Infectious Diseases Unit, Department of Internal Medicine, Fondazione IRCCS Ca' Granda Ospedale Maggiore Policlinico, Milan, Italy; 2 Department of Health Sciences (DISSAL), University of Genoa, Genova, Italy; 3 Infectious Diseases Clinic, Policlinico San Martino Hospital, Genoa, Italy; 4 Infectious Diseases Department, San Raffaele Scientific Institute, Milan, Italy; 5 Infectious Disease Unit, ASST Papa Giovanni XXIII, Bergamo, Italy; 6 Department of Informatics Bioengineering, Robotics, and Systems Engineering (DIBRIS), University of Genova, Genoa, Italy; 7 Infectious Diseases Unit, Department of Internal Medicine, Fondazione IRCCS Ca' Granda Ospedale Maggiore Policlinico, University of Milan, Milan, Italy; Institut Hospital del Mar d'Investigacions Mediques, SPAIN

## Abstract

Tenofovir alafenamide (TAF) has similar efficacy compared to tenofovir disoproxil fumarate (TDF), but a less favorable effect on lipids. Aim of this retrospective multicentre study was to evaluate the impact on lipids of switching from rilpivirine (RPV)/ emtricitabine (FTC)/TDF to RPV/FTC/TAF in a cohort of HIV-1 infected patients. Total cholesterol (TC), high density lipoproteins (HDL) and low density lipoproteins (LDL) were compared at the moment of the switch and at the first following evaluation, by using paired t-test. Overall, 573 patients were considered, 99% with HIV-RNA <50 copies/ml, with mean age of 49.7 (±0.4) years and median 13.4 (6.9–22.5) years of HIV infection. In the study population with available data (431/573, 75%), mean TC changed from 173 ±1.7 to 188 ±1.8 mg/dl; mean HDL from 46 ±0.7 to 51± 0.7 mg/dl; mean LDL from 111 ±1.5 to 120 ±1.8 mg/dl (p<0.0001 for all). Neither LDL/HDL nor TC/HDL ratio changed significantly, with LDL/HDL from 2.6 ±0.5 to 2.5 ±0.5 (p = 0.12) and TC/HDL from 4.0 ±0.6 to 3.9 ±0.6 (p = 0.11). In patients with baseline diagnosis of hypercholesterolemia (TC>200 mg/dl, N = 87), there was no significant change in TC (224 ±2.2 to 228 ±3.4 mg/dl, p = 0.286) or LDL (150±2.5 to 151±3.2 mg/dl, p = 0.751), while HDL increased from 51 ±1.6 to 55 ±1.7 mg/dl (p<0.0001) and both LDL/HDL and TC/HDL ratio decreased significantly, from 3.2±0.1 to 3.0 ±0.1 (p = 0.025) and from 4.7±0.1 to 4.4 ±0.1 (p = 0.004). In this real life study, a slight increase in lipids was found after switching from RPV/FTC/TDF to RPV/FTC/TAF, but these results were not confirmed in people with hypercholesterolemia, in which lipids did not change and LDL/HDL and TC/HDL ratio decreased.

## Introduction

Tenofovir disoproxil fumarate (TDF) has demonstrated high efficacy in all stages of HIV infection and in different drug combination regimens [1]. However, due to the progress of antiretroviral therapy (ART), new challenges for effective treatment of HIV have risen, focusing on safety and durability without threatening its efficacy. The introduction in the clinical practice of a new prodrug of tenofovir, tenofovir alafenamide (TAF), has allowed to reduce the bone and renal impact of TDF, but preserving its efficacy, thanks to a new formulation that minimize the release of the active tenofovir drug in plasma [2]. On the other side, these same benefits do not apply to the lipid effects of TAF that does not conserve the favorable profile that has been observed with TDF [3,4]. Rilpivirine (RPV) has been shown to have low metabolic impact as well, and could be a suitable companion for TAF due to its neutral profile on lipids [5–7]. Moreover, RPV also satisfies the needs of modern ART, being a well-tolerated and durable regimen, available in single tablet regimen co-formulated with TDF and emtricitabine (RPV/FTC/TAF) or with TAF and emtricitabine (RPV/FTC/TAF) [8–10]. A randomized, controlled, double-blind, Phase 3 trial evaluated the switching strategy from RPV/FTC/TDF to RPV/FTC/TAF in virologically suppressed patients. At week 96, switching to RPV/FTC/TAF was non inferior, in terms of virological suppression, but the switch resulted in increased total cholesterol (TC), low density lipoprotein (LDL), high density lipoprotein (HDL) and triglycerides (TG) [11]. However, no clinically significant change in TC/HDL ratio was noticed [11]. To date, there are still few data about the real life effects of switching from TDF to TAF, especially in the context of dyslipidemia. In a retrospective observational study of 384 patients who received elvitegravir/cobicistat/FTC/TDF (151 patients) or elvitegravir/cobicistat/FTC/TAF (233 patients), a significant increase in TC and LDL in naïve patients, and in TC, LDL, HDL, TC/HDL, LDL/HDL and TG in experienced patients, were observed after 48 weeks of TAF, while all lipids levels remained stable in the TDF arm [12]. In addition, the number of lipid-lowering drugs prescribed to patients who received TAF (11.9%) was almost triple compared to TDF (4,7%) [12]. The aim of this observational retrospective study was to evaluate the impact on lipids of switching from RPV/FTC/TDF to RPV/FTC/TAF single tablet regimen in a real life context and to investigate if the effects on lipids were different in people with different baseline values of TC and TG.

## Material and methods

This is a retrospective, observational, multicentre study, conducted in two Infectious Disease Centres in Northern Italy, ASST Papa Giovanni XXIII Hospital in Bergamo and Policlinico S. Martino Hospital in Genoa. Data for Policlinico S. Martino were retrieved from MedInfo, an online database for anonymous and automatic data collection [13]. Data from ASST Papa Giovanni XXIII Hospital Bergamo were obtained from Galileo, an on-line CRF that allows an anonymous data extraction. Data were afterwards merged in a single file for analysis. All patients who switched from RPV/TDF/FTC to RPV/TAF/FTC were included. We evaluated the change after the switch of lipid profile (TC, HDL, LDL and TG), CD4+ (CD4) and CD8+ T-lymphocytes (CD8) and creatinine, with two points evaluation: at baseline, before the switch, and at the first following evaluation. The evaluation of TC, HDL and LDL changes were investigated in all patients and then, separately, in those with or without baseline hypercholesterolemia (HC), defined as TC > 200 mg/dl. The evaluation of TG change was performed in all patients and then, separately, in those with or without baseline hypertriglyceridemia (HT), defined as TG > 200 mg/dl. The change of CD4 count was evaluated in all patients and in those with baseline CD4<500 cells/mm^3^.

Baseline was considered the day of the switch or the nearest blood exam preceding that date. The second timepoint was the first available evaluation immediately successive to the switch. Repeated evaluation performed after less than 6 weeks were excluded. Missing LDL were calculated with Friedewald equation [14]. HIV-1 RNA values were obtained by Versant HIV-1 RNA 1.0 Assay (kPCR) (Siemens HealthCare Diagnostics).

Continue variables were expressed as mean (± standard error, SE) or median (interquartile range, IQR), while categorical variables were expressed as frequencies. At baseline, a comparison between patients with or without HC was performed using unpaired two-samples t-test for variables with normal distribution (age, CD4, CD8, TC, HDL, LDL and creatinine) and by using Mann-Whitney U test for skewed variables (TG, TC/HDL, LDL/HDL, CD4/CD8 ratio, years of HIV infection). Categorical variable were compared at baseline between non-HC and HC patients by Chi squared test (female sex, risk factor for HIV) or by Fisher’s exacts test (frequency of baseline HIV-RNA >50 copies/mm^3^).

Data with normal distribution (CD4, CD8, TC, HDL, LDL and creatinine) were subsequently compared in the same subjects at baseline and follow up through paired t-test, while data with skewed distribution (TG, LDL/HDL, TC/HDL and CD4/CD8 ratio) were compared using paired samples Wilcoxon test. Changes in TC, LDL, HDL, LDL/HDL and TC/HDL were compared in patients with and without HC by using unpaired two-samples t-test, while changes of TG were compared in patients with or without HT by using Mann-Whitney U test.

A linear regression model was used to evaluate correlations between baseline values of TC, LDL, HDL and TG and their changes at follow up, that were expressed with regression coefficients, R, and 95% Confidence Intervals, 95%CI. Chi squared test was used to compare the frequencies of HC and HT before and after the switch.

All data were collected from December 2017 to April 2018. All patients followed in both Policlinico S. Martino Hospital and ASST Papa Giovanni XXIII Hospital signed an informed consent form in which they agreed use of their clinical data, in anonymous form, for scientific purposes. The use of the Ligurian HIV Network database for scientific purposes was approved by the Ligurian Ethics Committee (Date of approval 28 August 2013). The study has been performed in accordance with the ethical standards laid down in the 1964 Declaration of Helsinki and its later amendments and in accordance with Italian national laws.

## Results

Overall, 573 patients had switched from RPV/FTC/TDF to RPV/FTC/TAF, of whom 145 (25%) were females. Patients had mean age of 49.7 (±0.4) years and a history of median 13.4 (6.9–22.5) years of HIV infection. Risk factors for HIV acquisition were sexual intercourse in 374 (65%) patients (238 heterosexual, 136 homosexual), intravenous drug use in 181 (32%) and other/unknown factors in 18 (3%). In the whole population who switched from RPV/FTC/TDF to RPV/FTC/TAF, baseline mean CD4 were 777 ±13.7 cells/mm^3^, and HIV-RNA was < 50 copies/ml in 99.5%. The general characteristics of the study population are given in Table 1.

**Table 1:** Baseline characteristics of the study population and comparison between patients with and without baseline hypercholesterolemia (HC).

	All		Non-HC		HC		
	N	%	N	%	N	%	p
**Enrolled patients**	573	100	344	60.0	87	15.2	
**Female**	145	25.3	120	34.9	25	28.7	0.589^§^
**Risk factor for HIV acquisition**							
Sexual	374	65.3	219	63.7	62	71.3	0.375^§^
Intravenous drug use	181	31.6	111	32.3	23	26.4	
Transfusion/vertical/unknown	18	3.1	14	4.0	2	2.3	
**HIVRNA>50 copies/mL at T0**	3	0.5	3	0.9	0	0.0	1.000^§§^
	**Mean or median**	**SE or IQR**	**Mean or median**	**SE or IQR**	**Mean or median**	**SE or IQR**	**p**
**Mean age, SE (years)**	49.7	0.4	50.1	0.6	50.0	0.9	0.934*
**Median years of HIV infection, IQR**	13.4	6.9–22.5	14.5	8.2–23.5	13.0	6.4–21.0	0.204**
**Mean CD4, SE (cells/mm**^**3**^**)**	777	13.7	758	17.4	803	31.6	0.232*
**Mean CD8, SE (cells/mm**^**3**^**)**	920	19.0	882	22.6	1009	45.4	0.012*
**Median CD4/CD8 ratio, IQR**	0.90	0.6–1.2	0.90	0.6–1.2	0.86	0.6–1.1	0.217**
**Mean TC, SE (mg/dl)**	173	1.7	160	1.3	224	2.1	<0.0001*
**Mean LDL, SE (mg/dl)**	111	1.5	101	1.4	150	2.4	<0.0001*
**Mean HDL, SE (mg/dl)**	46	0.7	45	0.7	51	1.6	<0.0001*
**Median TG, IQR (mg/dl)**	98	75–147	94	74–139	122.5	91–188	<0.0001**
**Median TC/HDL ratio, IQR**	3.8	3.0–4.8	3.7	2.9–4.5	4.7	3.7–5.5	<0.0001**
**Median LDL/HDL ratio, IQR**	2.5	1.8–3.2	2.3	1.7–3.0	3.2	2.5–3.9	<0.0001**
**Mean creatinine, SE (mg/dl)**	0.96	0.01	0.97	0.01	0.97	0.01	0.832*

^§^ p-value calculated by Chi squared test

^§§^ p-value calculated by Fisher’s exact test

*p-value calculated by unpaired two-samples t-test

** p-value calculated by Mann-Whitney U test

SE: standard error; IQR: interquartile range; HC: hypercholesterolemia; TC: total cholesterol; LDL: low density lipoproteins; HDL: high density lipoproteins; CD4: CD4+T-lymphocytes; CD8: CD8+T-lymphocytes.

After a median follow up of 12 (8–24) weeks, although mean CD4 and CD8 remained stable, (CD4 from 777 ±13.7 to 787 ±14.5 cells/mm^3^, p = 0.4 and CD8 from 920 ±19.0 to 916 ±20.0 cells/mm^3^, p = 0.6, respectively), mean CD4/CD8 ratio slightly increased (from 0.96 ±0.02 to 0.99 ±0.02, p = 0.04). When limiting the analysis to patients with baseline CD4<500 cells/mm^3^ (N = 103, 18%), both CD4 and CD8 increased significantly from 368 ±9.2 to 412 ±12.8 cells/mm^3^ (p<0.0001) and, from 747 ±35.5 to 787 ± 39.5 cells/mm^3^, p = 0.037, respectively. CD4/CD8 ratio increased slightly, but significantly, in this group, from mean 0.6 ±0.03 to 0.7 ±0.04, p = 0.038.

Three patients (0.5%) with baseline HIV-RNA >50 copies/mL (median 358 copies/mL, IQR 209–54 copies/mL) achieved HIV-RNA < 50 copies/mL at follow up. Conversely, nine patients (1.5%) with baseline undetectable viremia had HIV-RNA >50 copies/mL at follow up (median 573 copies/mL, IQR 87–5,296 copies/mL).

In the same time frame the serum creatinine levels slightly but significantly decreased, from 0.96 (± 0.01) to 0.92 (± 0.01) mg/dL, p<0.0001.

**Fig 1:**
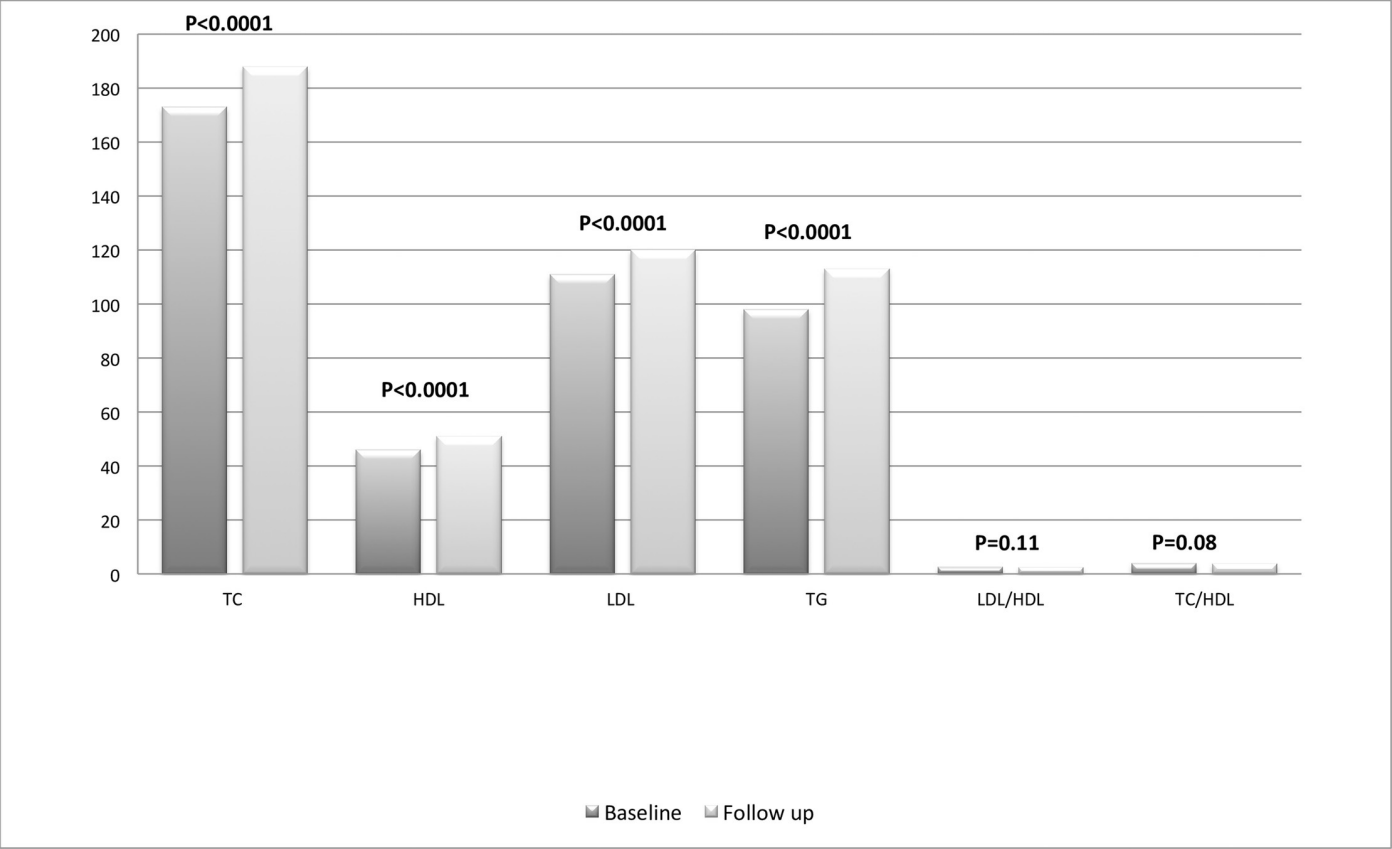
Change in hematic lipids across median 12 (8–24) weeks of follow up after switching from rilpivirine/emtricitabine/tenofovir disoproxil fumarate to rilpivirine/emtricitabine/tenofovir alafenamide. TC: total cholesterol, HDL: HDL cholesterol; LDL: LDL cholesterol, TG: triglycerides; IQR: interquartile range.

The evaluation of TC at both baseline and follow up was available for 431 out of the 573 patients (75%), HDL for 426 (74%), LDL for 423 (74%), TG for 430 (75%). At baseline, 45 patients were on statin therapy, and no patient added or stopped a lipid-lowering agent during the observation period. All lipids increased significantly (Fig 1): mean TC changed from 173 (±1.7) to 188 (±1.8) mg/dl; mean HDL from 46 (±0.7) to 51(±0.7) mg/dl; mean LDL from 111 (±1.5) to 120 (±1.8) mg/dl and median TG from 98 (75–147) to 110 (79–155) mg/dl, (p<0.0001 for all). Neither LDL/HDL nor TC/HDL ratio changed significantly, with LDL/HDL from 2.6 (±0.05) to 2.5 (±0.05), p = 0.12 and TC/HDL from 4.0 (±0.06) to 3.9 (±0.06), p = 0.11.

During the study period, the proportions of subjects with HC and HT increased, the first one from 20.1 to 36.4% (p<0.0001) and the second from 11.4 to 13.0% (p<0.0001).

Patients who developed HC had baseline TC levels slightly higher compared to patients who maintained normal TC levels [median 182 (170–192) vs 155 (138–172) mg/l, p<0.0001].

### Patients with baseline hypercholesterolemia (HC) or hypertriglyceridemia (HT)

When analyzing separately patients with baseline diagnosis of hypercholesterolemia (HC, N = 87, 20%), the change in TC and LDL resulted significantly different from that of other patients (non-HC, N = 344, 80%), p<0.0001 for both, Table 2. In fact, in patients with HC, TC did not change significantly, (from 224±2.2 to 228±3.4 mg/dl, p = 0.286), as well as LDL (from 150 ±2.5 to 151 ±3.2 mg/dl, p = 0.751). On the contrary, HDL increased significantly (from 51 ±1.6 to 55 ±1.7 mg/dl, p<0.0001), showing a similar trend to that observed in patients without HC (p = 0.173, Table 2).

**Table 2:** Changes across median 12 (8–24) weeks of follow up in total cholesterol (TC), HDL cholesterol (HDL), LDL-cholesterol (LDL), LDL/HDL ratio, TC/HDL ratio and triglycerides (TG), according to diagnosis of hypercholesterolemia (HC) or hypertriglyceridemia (HT) at baseline.

	**HC**	**Non-HC**	p*
TC (mg/dl)	+3.5 (±3.2)	+17.5 (±1.4)	<0.0001
HDL (mg/dl)	+3.4 (±0.1)	+4.8 (±0.4)	0.173
LDL (mg/dl)	+1.0(±3.1)	+11.3 (±1.2)	<0.0001
LDL/HDL	-0.2 (±0.1)	-0.01 (±0.3)	0.011
TC/HDL	-0.3(±0.1)	-0.01 (±0.04)	0.005
	**HT**	**Non-HT**	p**
TG (mg/dl)	-25 (-107;+28)	+10 (-9;+38)	<0.0001

Values are expressed as mean (±standard error) or median (interquartile range).

p* were calculated by using unpaired two-samples t-test

p** was calculated using Mann-Whitney U test

Both LDL/HDL ratio (from 3.2±0.1 to 3.0±0.1, p = 0.025) and TC/HDL ratio (from 4.7±0.1 to 4.4±0.1, p = 0.004) reduced significantly in patients with HC, and the reduction of both was more marked than that seen in other patients (p = 0.011 and p = 0.005, Table 2).

On the other side, in patients without baseline HC, TC changed from 161 ±1.3 to 178 ±1.8 mg/dl, LDL from 101 ±1.3 to 112 ±1.7 mg/dl, HDL from 45 ±0.71 to 50 ±0.82 mg/dl (p<0.0001 for all), while LDL/HDL ratio (from 2.4±0.05 to 2.4±0.05, p = 0.59) and TC/HDL ratio (from 3.8 ±0.06 to 3.8 ±0.06, p = 0.86) did not change significantly.

**Fig 2:**
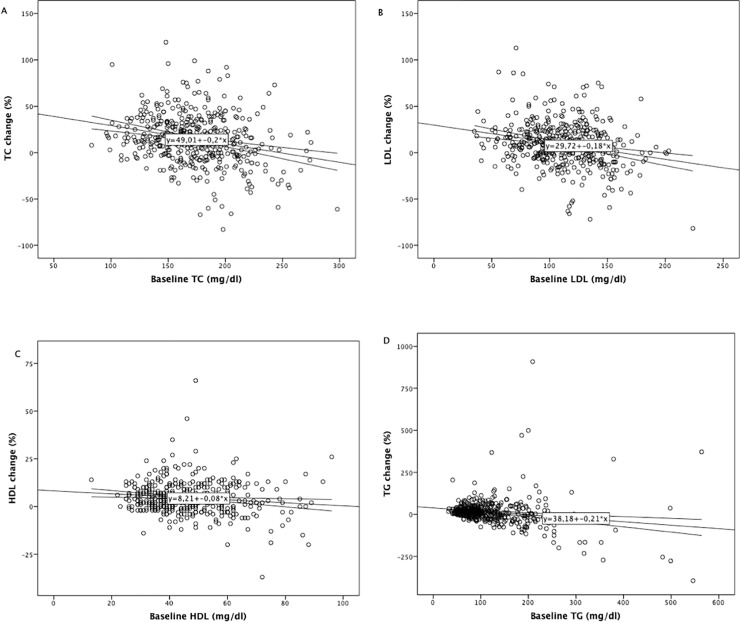
Correlations between total cholesterol (TC) change and baseline TC levels (A), LDL cholesterol (LDL) change and baseline LDL levels (B), HDL cholesterol (HDL) change and baseline HDL levels (C), triglycerides (TG) change and baseline TG levels (D).

The variation in TC resulted inversely correlated to baseline TC value (R -0.252, 95%CI -0.27; -0.13, p <0.0001, Fig 2A), as well as LDL change to LDL baseline value (R-0.238, 95%CI-0.26;-0.11, p<0.0001, Fig 2B) and HDL change to baseline HDL value (R-0.13, 95% CI-0.14;-0.02, p = 0.009, Fig 2C).

Patients who had baseline diagnosis of hypertriglyceridemia (HT, N = 50) did not experience significant change in TG levels during the study period, from median 238.5 (215–310) mg/dl to 212 (163–292) mg/dl, p = 0.088. TG trend was opposite to that seen in non-HT patents, with median -25 (-107;+28) mg/dl in HT vs. + 0 (-9;+38) mg/dl in non-HT patients (p<0.0001, Table 2). TG change at follow up inversely correlated to baseline TG levels (R-0.18, 95%CI -0.31;-0.10, Fig 2D).

## Discussion

The present study provides one of the first experiences in real clinical practice studying lipid profile, CD4 and creatinine changings in patients being switched from RPV/FTC/TDF to RPV/FTC/TAF. Our study showed three main findings. First, TC, HDL and LDL increased after the switch in patients without HC, while in HC patients these lipids showed a different trend, without significant variations in TC and LDL, but with decrease of TC/HDL and LDL/HDL ratio and increase of HDL. Second, CD4 increased significantly in patients with baseline CD4<500 cells/mm^3^. Third, creatinine levels decreased slightly, but significantly, after switching to RPV/FTC/TAF.

When considering all the study population, we found a significant increase in TC, HDL, LDL and TG in patients that have been switched from RPV/FTC/TDF to RPV/FTC/TAF, without significant variations in either LDL/HDL nor in TC/HDL ratios. These results reflected the main trend of lipids that have been previously observed in clinical trials [15]. However, the trend of lipids was not uniform in the study population. In fact, in patients who were diagnosed with HC at baseline, TC and LDL did not change significantly during the study. More importantly, in our study patients with HC experienced a significant increase in HDL levels and a decrease of LDL/HDL and TC/HDL ratios. The reduction of both these ratios has been previously found to predict a decrease in the risk of a first ischaemic heart disease, and TC/HDL ratio represents an important cumulative index of an atherogenic dyslipidemic profile associated with insulin resistance [16]. Thus, our results highlight the safety of the switch strategy from TDF to TAF, not only in patients without HC, where TC/HDL ratio remained unchanged, but also in patients with HC, which are those with the greatest need of therapies with low metabolic impact and that even experienced a reduction of TC/HDL ratio and an increase in HDL, both favoring the reduction of cardiovascular risk [16]. Also patients with baseline HT were studied. TG did not change significantly in this group, although they had a decreasing trend, differently from what found in the population without a diagnosis of HT. Taken together, our findings suggest that the representation of trends of TC, HDL and LDL, without distinguishing which patients have normal or high baseline TC value, could not be actually informative of what happens in the switch from TDF to TAF. In fact, the lipid increase is not consistent in all subjects, and is higher in people with lower baseline levels, namely those with the lowest cardiovascular risk. An inverse correlation between baseline levels of lipids and lipid increases was consistently confirmed for TC, LDL, HDL and TG levels in our study.

Of note, in our cohort, all patients were treated with the same fixed drug combination, also including RPV, a drug that has favorable metabolic profile [5,6]. Thus, it is possible that the same results could not be reproduced in patients taking TAF combined with less lipid-friendly antiretroviral agents. Moreover, despite our data are partially reassuring on the metabolic impact of TAF in patients with HC, it is worth of note that the frequency of HC and HT increased significantly in the study population, namely that some patients with normal TC at the beginning of the study, become HT after the switch to TAF, albeit without change in the LDL/HDL and TC/HDL ratios. This confirms the importance of TC monitoring, especially in patients who have significant cardiovascular risk factors.

The second important point arose from the study is the increase in CD4 seen in patients with baseline levels below 500 cells/mm^3^, after the switch to TAF. A possible contribution of TDF in the maintenance of low CD4 had been previously hypothesized in studies of dual therapy, where, after the removal of TDF, CD4 cells increased significantly [4]. However, these results were not confirmed in clinical trials studying the effect of the switch from TDF to TAF [11]. Nonetheless, it is possible that only patients with lower CD4 counts experience a significant increase, as the CD4 recovery is typically steeper in the first phases of treatment, and then, after a consistent increase, slows down till reaching a plateau [17]. Also in our study, the impact on CD4 was not relevant when considering the whole population and only the sub-analysis on patients with lower CD4 revealed a significant effect. Moreover, we do not have enough data to state if this effect can be linked to a cytopenic effect of TDF, to the prolongation of cumulative time on ART, or to a beneficial anti-inflammatory effect of TAF, that could even be hypothesized on the basis of the improvement in CD4/CD8 ratio that we observed in the study.

Finally, the third point investigated by our study was the effect of switching from RPV/FTC/TDF to RPV/FTC/TAF on creatinine levels. This study confirmed, in a real life context, a significant decrease of creatinine levels, in accordance with data from clinical trials on the renal safety of TAF [11].

The present study has several limitations. First, the retrospective, non-randomized, nature of the study confers low potency to its findings and information on change in lifestyle habits has not been evaluated. Moreover, only patients that have been switched from RPV/FTC/TDF to RPV/FTC/TAF have been included in the study, and thus the results might not be generalizable to other TAF containing ART regimens. Also, the prevalence of hypercholesterolemia that we found in our study, i.e. 20%, is lower than that estimated in the Italian population of people living with HIV, where up to 50% have been reported to have dyslipidemia [18]. Thus, the study population might not be representative of the Italian population living with HIV and this further limits the generalizability of our results. Additionally, the short follow-up of the study did not allow defining if the changings in TC, LDL, HDL, LDL/HDL and TC/HDL were confirmed in the long term, or if lipids levels become stable after a rapid change in the first weeks following the switch. Finally, we could not draw major conclusion on renal safety of the regimen, as we only had available creatinine evaluation after the switch, without data on other parameters of renal function such as or cystatin C values, albuminuria, or signs of proximal renal tubular impairment (low molecular weight proteinuria, hypophosphatemia/hyperphosphaturia, normoglycemic glycosuria, and hypouricemia/hyperuricuria).

## Conclusions

The present study highlight that, although a slight increase in TC occurs after switching from RPV/FTC/TDF to RPV/FTC/TAF, LDL/HDL and TC/HDL did not change in patients without HC, and even decreased in patients with HC. Moreover, patients with HC experienced a significant increase in HDL after being switched to RPV/FTC/TAF, and neither TC nor LDL changed significantly in them. Our research suggests that concerns about lipid alterations in patients with HC should not preclude TAF use in patients who need it for clinical reasons such as renal or bone diseases [3]. Future studies, focusing on actual cardiovascular risk and not only on lipid changes are desirable in such context, and stratification of results by baseline risk factors might be beneficial to better define which patients could have the better advantage from TAF use or, vice versa, which one would need tighter follow up or additional interventions. Further research will clarify the future impact of TAF in co-formulation with RPV on long-term metabolic and immunologic outcomes of people living with HIV.

## Supporting information

S1 Dataset (XLS)
